# Carriers of *POLG1* variants require investigations for multisystem disease and for mtDNA variations

**DOI:** 10.1186/s42466-022-00195-8

**Published:** 2022-07-11

**Authors:** Josef Finsterer, Sinda Zarrouk

**Affiliations:** 1 Neurology and Neurophysiology Center, Postfach 20, 1180 Vienna, Austria; 2 University of Tunis El Manar, Tunis, Tunisia

**Keywords:** mtDNA, POLG1, Multisystem disease, Mitochondrial, Lactate

Letter to the Editor

We read with interest the article by Dohrn et al. about four carriers of the novel, heterozygous *POLG1* variant c.3542G>A (Ser1181Asn) in a single family [1]. Phenotypically, the variant was reported to manifest with ptosis, ophthalmoplegia, myopathy of proximal limb muscles, and axonal neuropathy [1]. Transmission of the variant followed an autosomal dominant trait [1]. It was concluded that in patients with a neuro-myopathic phenotype an underlying *POLG1* variant should be considered [1]. The study is appealing but raises concerns that should be discussed.

The main limitation of the study is that sequencing of the mitochondrial DNA (mtDNA) had not been carried out in any of the four patients carrying the *POLG1* variant. Sequencing of the mtDNA is necessary to assess if the *POLG1* variant had any effects on the mtDNA, particularly if any secondary single or multiple mtDNA deletions occurred, which could have been responsible for the phenotype [2]. Additionally, it is crucial to assess by real time polymerase chain reaction (PCR) or non-real time PCR if the mtDNA copy number was normal or reduced. Reduction of mtDNA copy number (mtDNA depletion) has been described as a consequence of *POLG1* mutations [3], mtDNA depletion due to *POLG1* variants may manifest as syndromic or non-syndromic mitochondrial disorder (MID). Syndromic MIDs due to *POLG1* associated mtDNA depletion include progressive external ophthalmoplegia (PEO) [2], Leigh syndrome [4], Alpers Huttenlocher disease [5], or hepato-cerebral syndrome [6].

A further limitation of the study is that despite muscle biopsy in patient II.2 no biochemical investigations of the muscle homogenate had been carried out. Knowing respiratory chain functions is not only crucial for interpreting histopathological and immune-histopathological findings but also to assess the biochemical impact of the c.3542G>A variant.

Since *POLG1* variants frequently manifest with multisystem disease, it is crucial that the four affected family members had been systematically investigated for cerebral, ophthalmologic, otologic, endocrine, cardiac, gastro-intestinal, or renal involvement. Since *POLG-1* variants can manifest with epilepsy, stroke-like episodes (SLEs), cognitive impairment, leukoencephalopathy, basal ganglia, changes, cerebral atrophy, cerebellar atrophy, or calcifications, it is crucial to perform a cerebral magnetic resonance imaging (MRI), magnetic resonance spectroscopy (MRS), and electro-encephalography (EEG) in each of the affected patients. Prospectively investigating these patients for multisystem involvement is crucial as some organs may be subclinically affected.

Missing is the determination of the serum and cerebral lactate, which can be elevated in patients carrying *POLG1* variants.

We should be told how “proximal muscle fatigability” was assessed.

Overall, the interesting study has some limitations that call the results and their interpretation into question. Clarifying these weaknesses would strengthen the conclusions and could enhance the study. Patients carrying *POLG1* variants require prospective investigations for multisystem involvement and examination of the mtDNA.

## Data Availability

All data are available from the corresponding author.
